# Non-equivalence of anti-Müllerian hormone automated assays—clinical implications for use as a companion diagnostic for individualised gonadotrophin dosing

**DOI:** 10.1093/humrep/dex219

**Published:** 2017-06-22

**Authors:** Stamatina Iliodromiti, Barbara Salje, Didier Dewailly, Craig Fairburn, Renato Fanchin, Richard Fleming, Hang Wun Raymond Li, Krzysztof Lukaszuk, Ernest Hung Yu Ng, Pascal Pigny, Teddy Tadros, Joseph van Helden, Ralf Weiskirchen, Scott M. Nelson

**Affiliations:** 1 School of Medicine, University of Glasgow, Level two New Lister Building, Glasgow Royal Infirmary, Glasgow G31 2ER, UK; 2 Service de Gynecologie Endocrinienne et Medecine de la Reproduction, Hopital Jeanne de Flandre, Centre Hospitalier Regional Universitaire, 2 Avenue Oscar Lambret, 59037 Lille, France; 3 GCRM Ltd, 21 Fifty Pitches Way, Glasgow G51 4FD, UK; 4 University of Paris-Ouest, Suresnes, 200 Avenue de la République, 92000 Nanterre, France; 5 Department of Obstetrics and Gynaecology, The University of Hong Kong, Queen Mary Hospital, Pokfulam Road, Hong Kong; 6 INVICTA Fertility and Reproductive Center, 10 Rajska St., 80-850 Gdansk, Poland; 7 Department of Obstetrics and Gynaecological Nursing, Faculty of Health Sciences, Medical University of Gdansk, Gdansk, Poland; 8 Department of Gynaecological Endocrinology, Medical University of Warsaw, Warsaw, Poland; 9 Laboratoire de Biochimie and Hormonologie, Centre de Biologie Pathologie, Centre Hospitalier Regional Universitaire, Lille, France; 10 Laboratory Diagnostic Center University Hospital RWTH Aachen, Pauwelsstraße 30, D-52074 Aachen, Germany; 11 Institute of Molecular Pathobiochemistry, Experimental Gene Therapy and Clinical Chemistry, RWTH University Hospital Aachen, Pauwelsstraße 30, D-52074 Aachen, Germany

**Keywords:** follitropin delta, AMH, companion diagnostic, automated assays, immunoassays, personalised medicine, controlled ovarian hyperstimulation, IPD meta-analysis.

## Abstract

**STUDY QUESTION:**

Can anti-Müllerian hormone (AMH) automated immunoassays (Elecsys^®^ and Access) be used interchangeably as a companion diagnostic for individualisation of follitropin delta dosing?

**SUMMARY ANSWER:**

The Access assay gives systematically higher AMH values than the Elecsys^®^ assay which results in over 29% of women being misclassified to a different follitropin delta dose.

**WHAT IS KNOWN ALREADY:**

Follitropin delta is the first gonadotrophin to be licenced with a companion diagnostic, the Roche Elecsys^®^ AMH Plus assay. Alternative automated AMH assays including the Beckman Coulter Access immunoassay are considered to provide similar results, but clarification of their suitability as an off-licence companion diagnostic for follitropin delta is required.

**STUDY DESIGN, SIZE, DURATION:**

We systematically searched the existing literature for studies that had measured AMH using both automated assays in the same cohort of women. Individual paired patient data were acquired from each author and combined with unpublished data.

**PARTICIPANTS/MATERIALS, SETTING, METHODS:**

We identified five eligible prospective published studies and one additional unpublished study. A 100% response from the authors was achieved. We collected paired AMH data on samples from 848 women. Passing–Bablok regression and Bland–Altman plots were used to compare the analytical performance of the two assays. The degree of misclassification to different treatment categories was estimated should the Access AMH be used as a companion diagnostic instead of the Elecsys AMH in determining the dosing of follitropin delta.

**MAIN RESULTS AND THE ROLE OF CHANCE:**

The Passing–Bablok regression shows a linear relationship (Access = −0.05 + 1.10 × Elecsys). The Access assay systematically gave higher values by an average of 10% compared with the Elecsys assay (slope = 1.10, 95% CI: 1.09 to 1.12). The average of the difference between the two assays was 2.7 pmol/l. The 95% limits of agreement were −11.7 to 6.3. Overall 253 (29.3%) women would have received an inappropriate follitropin delta dose if the Beckman Coulter Access assay was used. Specifically, a substantial proportion of women (ranging from 49% to 90% depending on the AMH category) would receive a lower dose of follitropin delta based on the Access AMH assay. Up to 10% (ranging from 2.5% to 10%) of women with high ovarian reserve would have been misclassified to a greater dose of follitropin delta based on the Access AMH assay.

**LIMITATIONS REASONS FOR CAUTION:**

We compared the values of the two principal automated assays, extrapolation of our findings to other automated AMH assays would require similar comprehensive examination.

**WIDER IMPLICATIONS OF THE FINDINGS:**

An international standard for the calibration of the automated AMH assays is warranted to facilitate efficient use of AMH as a companion diagnostic. The variable calibration of alternative automated AMH assays may adversely impact on the performance of the follitropin delta dosing algorithm.

**STUDY FUNDING/COMPETING INTEREST(S):**

No formal funding has been received for this study. SI is funded by a UK Medical Research Council skills development fellowship (MR/N015177/1). SMN has received speakers fees, travel to meetings and participated in advisory Boards for Beckman Coulter, IBSA, Ferring Pharmaecuticals, Finox, Merck Serono, Merck and Roche Diagnostics. SMN has received research support from Ansh laboratories, Beckman Coulter, Ferring Pharmaceuticals and Roche Diagnostics.

**TRIAL REGISTRATION NUMBER:**

N/A.

## Introduction

Personalised stimulation protocols for assisted conception that account for patients’ characteristics and prognostic biomarkers to attain an ovarian optimal response have been the clinical vision in reproductive medicine (Nelson, 2013; La Marca and Sunkara, 2014; Iliodromiti *et al.*, 2015). In other clinical fields, the use of a companion diagnostic has been the hallmark of personalised medicine, and formally extends the role of a specific biomarker, measured in an explicit way, to guide treatment. The Food and Drug Administration (FDA) and European Medicines Authority (EMA) define a companion diagnostic as a medical device, often an *in vitro* device, which provides information that is essential for the safe and effective use of a corresponding drug or biological product (FDA, 2017). As such the use of an explicit companion diagnostic with a particular therapeutic product is stipulated in the instructions for use in the labelling of both the diagnostic device and the corresponding therapeutic product. This is to ensure that a result provided by an alternative provider does not result in treatment decisions that may not be optimal.

A recent randomised controlled phase 3 trial has confirmed the efficacy of individualised dosing of the gonadotrophin, follitropin delta, for controlled ovarian hyperstimulation. Pharmacokinetic and pharmacodynamic simulation facilitated development of an individualised dosing algorithm for follitropin delta incorporating body weight, which influences drug exposure, and pre-treatment anti-Müllerian hormone (AMH) levels, which predict ovarian response. The dosing algorithm is specific for follitropin delta and is designed to maintain ongoing pregnancy rates and reduce the risk of extreme ovarian responses (Nyboe Andersen *et al.*, 2017). AMH as determined on the Roche Elecsys automated platform was a critical component of the individualised dosing algorithm, led to this assay being incorporated into the licencing of this novel drug as the specified companion diagnostic.

The optimal performance and stability of automated AMH assays over previous manual assays is well recognised (Li *et al.*, 2016; Pigny *et al.*, 2016), with many clinicians assuming that the values derived from the two most common automated assays, namely, the Elecsys^®^ AMH assay (Roche Diagnostics International Ltd) and the Access AMH assay (Beckman Coulter Diagnostics) are interchangeable. Small single-centred studies have demonstrated almost identical values of AMH in the same samples measured in both assays (Tadros *et al.*, 2016), while others have suggested that the Access determined AMH values were 5–15% higher than that measured by the Elecsys system (Nelson *et al.*, 2015; Li *et al.*, 2016; Pigny *et al.*, 2016). It is unclear whether evidence of disagreement between the two diagnostic methods may increase with larger sample number, with a wider distribution of AMH and whether this discordance has any clinical implications. The aims of our study were to summarise the available evidence, compare the performance of the two automated assays in a wider range of women and determine whether the Access AMH assay would have stratified women to different dosing categories of follitropin delta compared with the Elecsys^®^ AMH assay, which is the licenced companion diagnostic of choice.

## Methods

### Data acquisition

We searched the literature for studies on AMH and automated assays. A systematic search was performed in Medline, Embase and Pubmed up to December 2016. Keywords used were synonyms for AMH (anti-Müllerian hormone or Müllerian inhibiting substance) and relevant to automated assays (automated assay or Elecsys^®^ or Access). All titles and abstracts were evaluated for eligibility by two authors (SI and SMN). Potentially eligible papers were read in full and relevant papers were selected. Eligible studies were studies that had measured AMH using both Elecsys^®^ and Access automated assays in the same cohort of women. Reviews and case reports were excluded. There was no language restriction. In the identified studies, AMH was measured in serum samples collected from women of reproductive age using standard procedures and cryopreserved before measurement. The assays were not necessarily performed simultaneously.

All authors of the selected papers were informed of the individual patient data (IPD) protocol and invited to share their data. After data acquisition, data were transferred into a single spreadsheet. Unpublished data (from personal communication) were included in the spreadsheet. AMH values were converted to pmol/l (ng/ml = 7.14 pmol/l). Inconsistencies and invalid values were checked with the original authors.

### Statistical analysis

Comparisons between the two methods were assessed using Bland–Altman plots and Passing–Bablok regression. The 95% limits of agreement in the Bland–Altman plot assume no bias between the two methods and were assessed by visualisation. Passing–Bablok regression was used to describe the relationship between the two methods with the Elecsys^®^ assay being considered as the gold standard as it has already been evaluated and licensed as the companion diagnostic for follitropin delta (Nyboe *et al.*, 2016). Passing–Bablok regression does not assume freedom of variables from error, nor does it make any assumptions about the distribution of the data and hence it may be less influenced by outliers. The percentage of women that would have been stratified to a different dose of follitropin delta based on the AMH value measured with the Access rather than the Elecsys^®^ assay were estimated. All statistics were performed using Stata v14.1 (StataCorp, Texas, USA).

## Results

**Figure 1:**
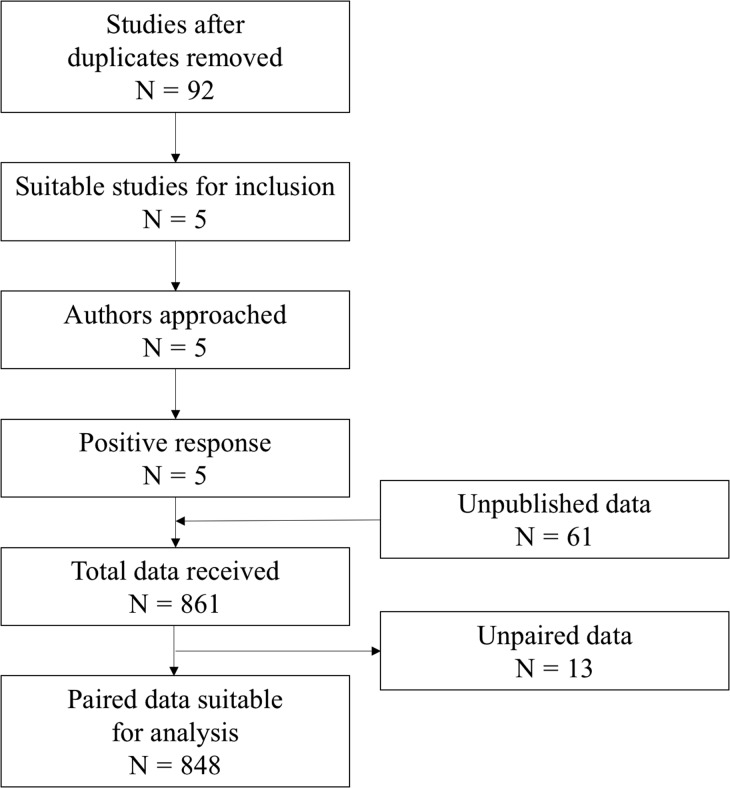
Flow chart of search strategy and data combination.

Figure 1 shows the flow chart of the search strategy. We selected five published studies (Nelson *et al.*, 2015; van Helden and Weiskirchen, 2015; Li *et al.*, 2016; Pigny *et al.*, 2016; Tadros *et al.*, 2016) that fulfilled our inclusion criteria. All authors responded to our request to share their raw data. We also included data from 61 women from personal correspondence with two of the co-authors (CF, RF). Supplemental data Table SI shows the main characteristics of the studies/cohorts included in our summary analyses.

**Figure 2:**
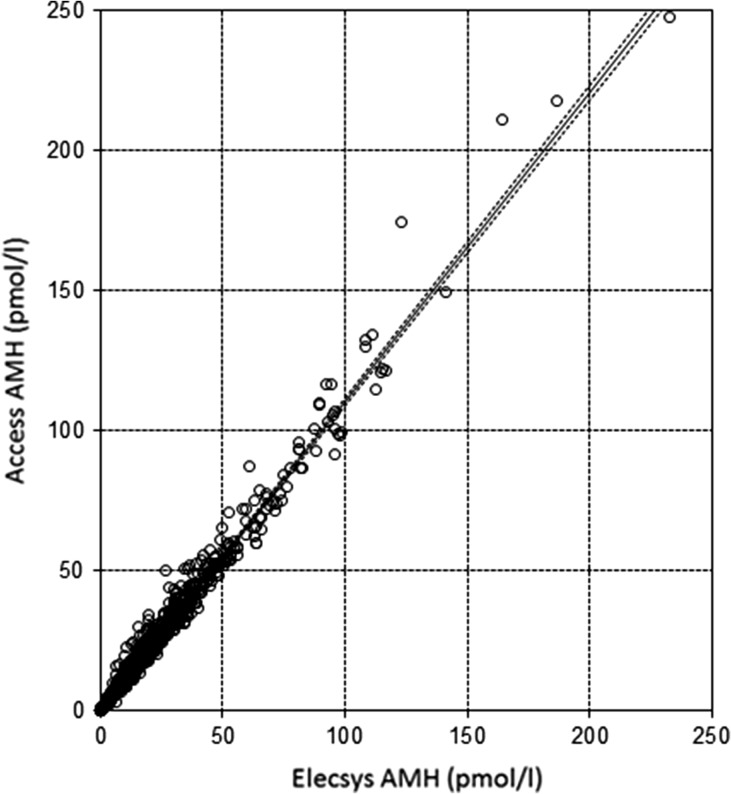
Passing–Bablok regression line with 95% confidence intervals of Elecsys and Access Assays. Passing–Bablok regression line with 95% confidence intervals Access = −0.05 + 1.10 (95% CI: 1.09–1.12) × Elecsys. The dots represent the paired values of AMH.

**Figure 3:**
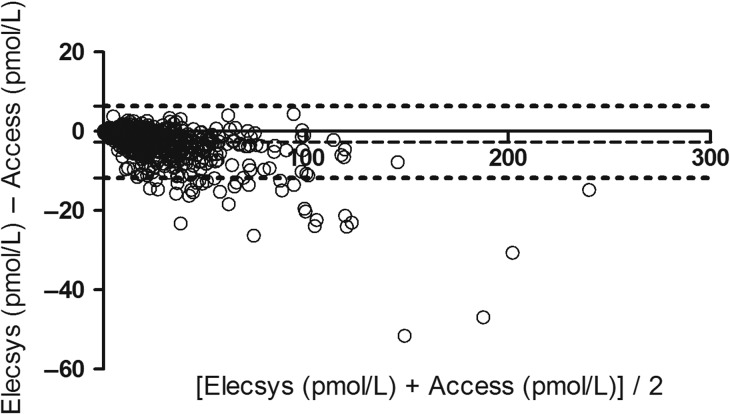
Bland–Altman plot of Elecsys and Access Assay. Mean difference −2.7 pmol/l (dashed line) with 95% confidence intervals (−11.7 to 6.3) (dotted lines).

In total, we had AMH values for 861 women, of whom 848 had paired values measured with both assays. Supplementary data Figure S1 demonstrates the distribution of AMH values for each assay, with median 14.9 pmol/l (IQR: 6.6–28.2, range 0.07–232.6) for Elecsys^®^ assay as compared to 17.0 pmol/l (IQR: 7.5–31.9, range 0.05–247.4) for Access assay (*P* < 0.001). Figures 2 and 3 show the Passing–Bablok regression and Bland–Altman plot comparing the two assays across the range of AMH values. The Passing–Bablok regression shows a linear relationship (Access = −0.05 + 1.10 × Elecsys) with minimal visual disparity between the two assays. However, the Access assay systematically gave higher values by an average of 10% compared with the Elecsys assay (slope = 1.10, 95% CI: 1.09–1.12). The Bland–Altman plot suggests that the discordance between the two assays was consistent with increasing values of AMH. On average, Access assay measured on average 2.7 pmol/l (bias) greater values than the Elecsys^®^ assay based on a median concentration of circa 15 pmol/l. The 95% limits of agreement were −11.7 to 6.3.

Table I presents the percentage of patients that would have been classified to a different treatment category of follitropin delta should the AMH have been measured with the Access assay instead of the Elecsys^®^ assay. In total, 253 (29.3%) women would have been classified to a different follitropin delta dose based on use of the Access assay as compared to the Roche assay. Specifically a substantial proportion of women (ranging from 50% to 90% depending on the AMH category) would have been given a lower dose of follitropin delta based on the Access assay. Although the overall values are higher with the Access assay, up to 10% (ranging from 2.9% to 10%) of women with high ovarian reserve would have been misclassified to a greater dose of follitropin delta based on the Access assay.

**Table I:** Follitropin delta dosing algorithm and risk of dosing misclassification.

Serum AMH (pmol/l) based on Elecsys assay^†^	Daily dose of Follitropin delta^†^	Number of women based on Elecsys assay (*N* = 848)	*N* (%) misclassified to greater treatment category (lower dose) based on Access assay^*^	*N* (%) misclassified to lower treatment category (higher dose) based on Access assay^*^
<15	12 μg	426	38 (8.9)	–
15–16	0.19 μg/kg	43	26 (60.4)	2 (4.7)
17	0.18 μg/kg	19	17 (89.5)	0 (0)
18	0.17 μg/kg	25	21 (84.0)	1 (4.0)
19–20	0.16 μg/kg	33	20 (60.6)	3 (9.1)
21–22	0.15 μg/kg	35	25 (71.4)	1 (2.9)
23–24	0.14 μg/kg	20	16 (80.0)	2 (10.0)
25–27	0.13 μg/kg	31	22 (71.0)	1 (3.2)
28–32	0.12 μg/kg	47	28 (59.6)	0 (0)
33–39	0.11 μg/kg	51	25 (49.0)	2 (3.9)
≥40	0.10 μg/kg	118	–	3 (2.5)

^†^Follitropin delta dosing algorithm as in provided in the prescribing information. All AMH values <15 pmol/l receive 12 μg irrespective of body weight in first cycle. For AMH values above ≥15 pmol/l the dose is per kg body weight.

^*^Number and % of women misclassified for each AMH dosing strata

## Discussion

The Elecsys^®^ AMH Plus immunoassay by Roche is the first companion diagnostic that has been approved for individualisation of gonadotropin dosing in reproductive medicine. In accordance with FDA and EMA guidance on companion diagnostics, this immunoassay is stipulated in the EMA licence of follitropin delta. Herein, we report evidence from a large sample of women (*n* = 848) with a wide distribution of AMH values in whom values obtained by an alternative automated AMH assay, the Beckman Coulter Access assay, were systematically higher than that by the Elecsys^®^ assay, by an average of 10%. This apparently modest discordance would result in an overall 29% of women being classified to an incorrect follitropin delta dose.

Reproductive medicine clinicians are accustomed to interpreting a range of biomarkers to guide clinical decision-making. However, the era of companion diagnostics formalises this relationship, with a specific companion diagnostic stipulated in the licencing and prescribing guidance of a drug. As such, regulatory authorities now recommend the joint development of therapeutic products and diagnostic devices (FDA, EMA 2017). As the results from the diagnostic device/test are essential for patient treatment, health care professionals must be able to rely on those results. Imprecise performance of an *in vitro* diagnostic (IVD) could have substantial therapeutic consequences, and lead to the administration of inappropriate therapy hence the strict drug licencing conditions with the specific IVD test named.

Individualised ovarian stimulation with follitropin delta relies on very narrow categories of AMH to stratify women to the appropriate gonadotropin dose to achieve a target ovarian response of 8–14 oocytes (Arce *et al.*, 2014; Bosch *et al.*, 2015; Nyboe Andersen *et al.*, 2017). That a large number of women would be classified differently if an alternative assay was used, in part reflects the small AMH categories that were used in the original dosing algorithm. Classifying women to a lower gonadotrophin dose because of difference in calibration of the Access AMH assay would likely lead to a systematic shift in the ovarian response to the left with the potential for inadequate follicular development and subsequent lower live-birth rates (Sunkara *et al.*, 2011; Steward *et al.*, 2014). Whereas, a small but clinically substantial proportion of women with high ovarian reserve may be stratified to receive a higher gonadotrophin dose based on the Access AMH assay, with an inherent increased risk of iatrogenic ovarian hyperstimulation syndrome or cycle cancellation (Steward *et al.*, 2014). Both scenarios, albeit requiring confirmation from off-licence studies examining the clinical implications of inappropriate follitropin delta dosing, counteract the value of biomarker-tailored personalised treatment with potential detrimental effects. In addition, both diminished ovarian response and iatrogenic hyperstimulation due to inappropriate follitropin delta dosing may be associated with financial implications for patients and state funders. Standardisation of the automated AMH assays or validation of the dosing algorithm to the technical characteristics of other automated assays is critical to ensure the successful transition to individualised stimulation protocols.

The underlying cause for the discordance between assays is unclear. The systematic difference across the range of AMH values and the association between the two assays suggest a discordance in calibration rather than assay technical characteristics or linearity. The Elecsys assay has been standardised via sample value transfer from the Gen II assay under the unmodified protocol using an aged serum panel (Gassner and Jung, 2014). The Access AMH assay was harmonised with the Gen II assay under the modified protocol using frozen samples (Demirdjian *et al.*, 2016). These AMH concentrations were then transferred to Access AMH mean signal counts to assign values to reference calibrators prepared with recombinant total AMH (Immunotech (IOT)) in HEPES buffer using three reagent pack lots (Demirdjian *et al.*, 2016). That the Access assay currently utilises a recombinant AMH for manufacturing of calibration standards, may potentially contribute to less batch to batch variability and maintenance of the observed discordance. An universal recombinant based standard adopted by all manufacturers for the calibration of the automated AMH assays will be critical to facilitate individualisation of ovarian stimulation. This will particularly be the case for follitropin delta, but will also apply to universal interpretation of AMH results for selection of alternative gonadotropin doses.

Our study has several strengths, it is the largest study comparing the technical performance of the two automated assays. We combined existing with unpublished data by implementing an IPD protocol to show the limits of agreements of the two assays in a wider range of AMH values than each single study. This range of clinically relevant values enabled us to provide a robust estimate of the discordance between the two assays that may not be detected in single centre studies examining specific sub-groups of women. The systematic search of the literature, the 100% response rate and inclusion of unpublished data minimised the risk of publication bias. This is the first study that evaluated the discordance in the two automated assays and its impact in dosing misclassification and underscored the need for universal standards in the calibrations of the automated assays. All included studies used a prospective design and implemented the manufacturer analytical guidance minimising the between study heterogeneity. Automated assays show substantial pre-analytical stability to different room temperatures, freezing and storage conditions (Demirdjian *et al.*, 2016) overcoming the limitation of inter-laboratory measurements. We do however acknowledge several limitations; we only compared two of the most frequently used automated assays rather than all of the available automated assays, but for many geographical areas including Europe, Australia and the USA the Biomerieux assay will not be available. We have not assessed the effect of the intra-assay coefficient of variation (CV), and accept that repeat measurements on the same sample may have resulted in misclassification, but the inter assay CV has been reported by several multi-centre studies as <5% (Gassner and Jung, 2014; Anckaert *et al.*, 2016). Undoubtedly, the need for universal calibration using international standards applies to all automated assays.

## Conclusion

The two most widely used automated AMH assays have modest but systematic differences in their values. The clinical implication of this is most striking for a novel individualised dosing algorithm based on a licenced companion diagnostic assay, with a large number of women at risk of inappropriate dosing if an alternative automated AMH assay is used.

## Supplementary data

Supplementary data are available at *Human Reproduction* online.

## Authors’ roles

SMN conceived the idea. SI performed the statistical analysis and wrote the initial draft. SI, BS and SMN performed the systematic search and SMN contacted the primary authors for the IPD analysis. All authors reviewed and accepted the final version of the manuscript.

## Funding

No formal funding has been received for this study. SI is funded by a Medical Research Council Medical Research Council skills development fellowship (MR/N015177/1).

## Conflict of interest

SMN has received speakers fees, travel to meetings and participated in advisory Boards for Beckman Coulter, IBSA, Ferring Pharmaecuticals, Finox, Merck Serono, Merck and Roche Diagnostics. SMN has received research support from Ansh laboratories, Beckman Coulter, Ferring Pharmaceuticals and Roche Diagnostics.

## Supplementary Material

Supplementary Data

Supplementary Data
