# Supplementary material for: Non-equivalence of anti-Müllerian hormone automated assays—clinical implications for use as a companion diagnostic for individualised gonadotrophin dosing
Source: Hum Reprod. 2017 Jun 22;32(8):1710–5. doi: 10.1093/humrep/dex219 (PMC5850658; doi:10.1093/humrep/dex219)
Supplement: Supplementary Data [file figures1.pdf]

Human Reproduction, 2017
doi:10.1093/humrep/dex219

human reproduction

SUPPLEMENTARY DATA

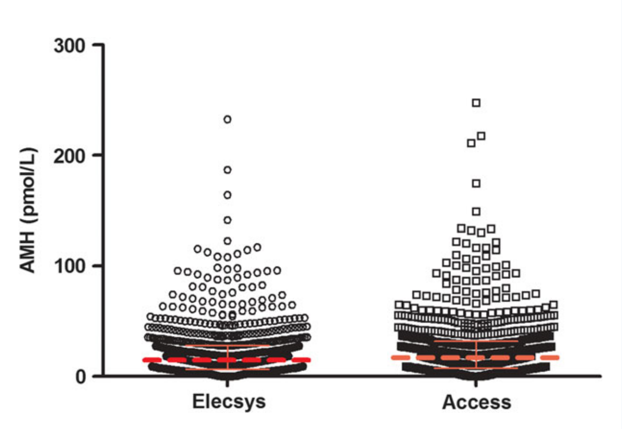

Figure S1 Distribution of AMH values measured with Elecsys and Access assay. Red lines represent median (dashed line) with inter-quartile range (solid lines).
