# Supplementary material for: Non-equivalence of anti-Müllerian hormone automated assays—clinical implications for use as a companion diagnostic for individualised gonadotrophin dosing
Source: Hum Reprod. 2017 Jun 22;32(8):1710–5. doi: 10.1093/humrep/dex219 (PMC5850658; doi:10.1093/humrep/dex219)
Supplement: Supplementary Data [file supplementaltablesi.pdf]

Supplemental Table SI **Characteristics of included studies**

Author year	Number of patients	Patient selection	Age range (years)	Assays compared	Storage	Sample taken	Notes
Nelson *et al.* (2015)	83	Assisted conception patients		Access<br>Elecsys<br>Gen II Assay<br>Ansh Labs	−80°C	Days 2–5 of cycle	
Li *et al.* (2016)	265	Assisted conception patients		Access<br>Elecsys<br>Gen II Assay	Fresh −20°C (2 weeks)<br>−80°C (2 weeks)	Not documented	Diagnostic performance for PCOS also compared.
Tadros *et al.* (2016)	211	Assisted conception patients	24–43	Access<br>Elecsys<br>Gen II Assay	Frozen thawed samples. No temperature given<br>Frozen for 2–4 months	Days 1–5 of cycle	Inclusion Criteria: Both ovaries present with no morphological abnormalities BMI 18–25 kg/m^2<br>Optimum ovarian visualisation at transvaginal sonography
van Helden and Weiskirchen (2015)	146	Assisted conception patients PCOS<br>Menopausal patients	19–53	Access<br>Elecsys<br>Gen II Assay	−30°C	Days 2–4 of cycle	Simultaneous AFC by transvaginal sonography
Pigny *et al.* (2016)	95	PCOS	18–35	Access<br>Elecsys<br>Gen II Assay<br>AL-105i<br>ELISAs<br>EAI AMH/MIS	−80°C	Days 2–5 of cycle (spontaneous or induced with dydrogesterone 10 mg/day for 7 days)	Exclusion criteria: Age <18 or >35<br>Suspicion of low ovarian reserve hyperprolactinaemia nonclassic 21-hydroxylasedeficiency<br>Ovarian/Adrenal tumours hypothalamic amenorrhoea follicle >9 mm/serum E_2 > 80 pg/ml<br>Unexplained infertility<br>Endometriosis
